# High-Efficiency Visible Light Manipulation Using Dielectric Metasurfaces

**DOI:** 10.1038/s41598-019-42444-y

**Published:** 2019-04-24

**Authors:** Rifat Ahmmed Aoni, Mohsen Rahmani, Lei Xu, Khosro Zangeneh Kamali, Andrei Komar, Jingshi Yan, Dragomir Neshev, Andrey E. Miroshnichenko

**Affiliations:** 1 Nonlinear Physics Centre, Research School of Physics and Engineering, The Australian National University, Canberra, ACT 2601 Australia; 2 School of Engineering and Information Technology, University of New South Wales, Canberra, ACT 2600 Australia

**Keywords:** Nanophotonics and plasmonics, Nanophotonics and plasmonics

## Abstract

The development of a miniaturised device that provides efficient beam manipulation with high transmittance is extremely desirable for the broad range of applications including holography, metalens, and imaging. Recently, the potential of dielectric metasurfaces has been unleashed to efficiently manipulate the beam with full 2π-phase control by overlapping the electric and magnetic dipole resonances. However, in the visible range for available materials, it comes with the price of higher absorption that reduces efficiency. Here, we have considered dielectric amorphous silicon (a-Si) nanodisk and engineered them in such a way which provides minimal absorption loss in the visible range. We have experimentally demonstrated meta-deflector with high transmittance which operates in the visible wavelengths. The supercell of proposed meta-deflector consists of 15 amorphous silicon nanodisks numerically shows the transmission efficiency of 95% and deflection efficiency of 95% at operating wavelength of 715 nm. However, experimentally measured transmission and deflection efficiencies are 83% and 71%, respectively, having the experimental deflection angle of 8.40°. Nevertheless, by reducing the supercell length, the deflection angle can be controlled, and the value 15.50° was experimentally achieved using eight disks supercell. Our results suggest a new way to realise the highly transmittance metadevice with full 2π-phase control operating with the visible light which could be applicable in the imaging, metalens, holography, and display applications.

## Introduction

During the last few decades, optics and photonics areas have demonstrated positive influence from every aspect of the modern nanotechnology. New nanofabrication techniques help to miniaturise optical devices to achieve fast response and cost-effectiveness comparing to the other electronics technologies^1^. Available conventional optical components are still bulky, costly, and difficult to manufacture with high accuracy which limits their applications, especially in portable and wearable devices^2,3^. Metasurfaces have shown outstanding abilities to overcome these issues through their unique light manipulation capabilities. Metasurfaces are an ultrathin single-layer planar nanostructures made of subwavelength metallic or dielectric elements which can be designed to efficiently control the light characteristics such as polarisation, dispersion, amplitude, and phase^4–6^. In particular, the efficient 2π-phase control capability with high transmittance feature makes the metasurfaces versatile tools for many applications including flat lenses, holograms, vortex generators, polarimeters, imaging, etc.^7–10^. Indeed, the 2π-phase response of the dielectric metasurfaces can be achieved via Mie-resonance, Pancharatnam-Berry phase elements and in-plane anisotropy of the scatterer^11–14^.

In this paper, we concentrate on Mie-resonance Huygens’ dielectric metasurfaces that take place by overlapping electric dipole (ED) and magnetic dipole (MD) resonances. Such metasurfaces can overcome the absorption loss of the materials leading to a full 2π-phase response with near-unity transmission^15,16^. Recently, Adam *et al*. reported the high-efficiency all-dielectric Huygens’ metasurfaces, which show the experimental refraction efficiency of 63.6% (infrared) and 78% (optical frequency)^17^. Zhou *et al*. reported the implementation of crystalline-silicon based dielectric metasurface with the experimental beam deflection of 19.27° and transmission efficiency of 67% at 532 nm wavelength^18^. Shalaev *et al*. also reported a rectangular nanopost based beam steering approach, which has a transmission efficiency of 36% at telecommunication wavelength^19^. In another paper, an all-dielectric C-shaped gradient metasurface was employed to achieve a deflection angle of 15° at the normal incident beam^20^. Furthermore, another interesting approach for electromagnetic wave manipulation was shown through elliptical disk based gradient metasurface leading to a wave deflection of 7°^21^. Chalcogenide alloy PbTeLi based high-efficiency transmittance metasurfaces was also reported with the experimental transmission efficiency of 75% and a deflection angle of 15.1° at mid-infrared wavelengths^22^. Utilising liquid-crystals, thermally-tunable dielectric metasurfaces was also reported, showing the dynamic beam switching from zero to 12° with 50% transmission efficiency^23^. Recently, Sell *et al*. showed nonintuitive geometry that can significantly improve the deflection angle and achieve the wide deflection angle of 75° with the total deflection efficiency of 75% (TM polarized mode) at the operating wavelength 1050 nm^24^. Nevertheless, this sophisticated geometry requires precise optimisation and its fabrication is challenging. Several theoretical approaches were also reported where transmission and reflection modes were used to achieve the deflection behaviour^25–27^. However, the bottleneck of all abovementioned techniques shows the low transmission of the individual unit cell in the visible range. Therefore, the overall transmission and deflection efficiency of the metadeflector in the visible range was diminished significantly^18,19^. As a result, there is a serious quest to improve the transmission and deflection efficiency of optical devices in the visible range. Silicon has been employed as a material of choice in the near and mid-infrared regions due to its negligible loss in these regions. However, it exhibits remarkable absorption/loss in the visible range.

In this work, we propose an engineered dielectric metadevice operating at visible wavelengths. We have designed and fabricated a silicon metasurface that exhibits not only an efficient beam deflection capability but also a high transmission property utilising the phase control. Our metasurface consists of circular nanodisk based supercells fabricated from amorphous silicon. By changing the diameter of the disks we manipulate the phase delay of the electromagnetic wave. Utilising properties of near-unity transmittance and discrete phase of dielectric nanodisks, we have demonstrated that the transmitted beam angle can be varied significantly by controlling the supercell length. Furthermore, we have introduced a way to combine the waves coming from two different supercells that leads to excitation of high diffraction orders.

## Results and Discussions

**Figure 1:**
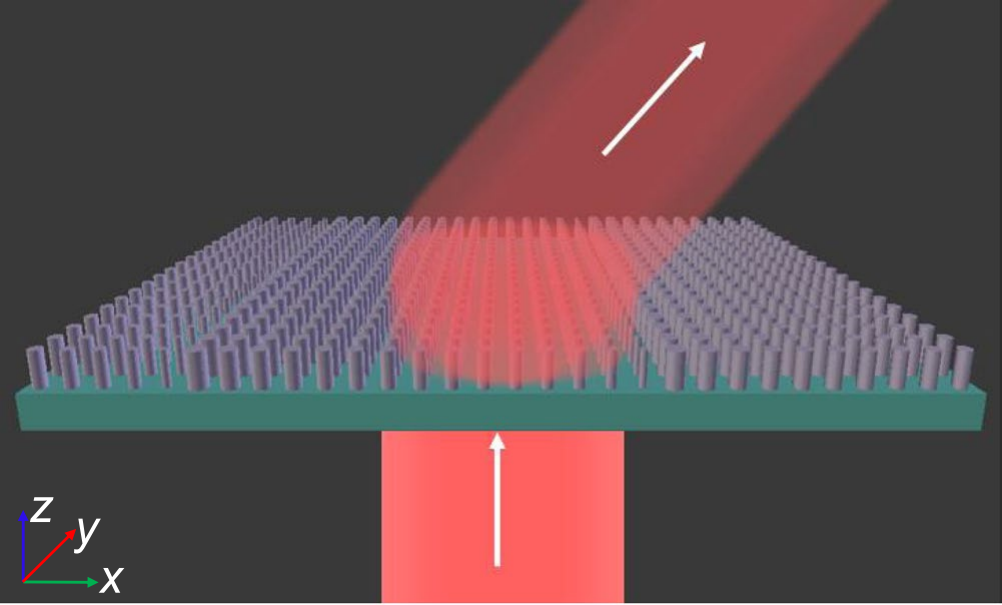
Schematic of the proposed beam deflector, with supercell containing fifteen nanodisks where each responsible for phase shift from 0 to 2π with π/7 increments.

We have designed and fabricated three different gradient metasurfaces, consisting of fifteen (2π phase response), eight (2π phase response) and fifteen (3π phase response) a-Si nanodisks that can efficiently manipulate the propagating wave angles. The schematic of the proposed fifteen disks supercells is shown in Fig. 1. The disks are arranged periodically in order of phase from 0 to 2π with π/7 increment, subsequently, which helps to bend the propagating wave following the phase delay phenomenon. The height (h) of the disks is 352 nm, and the disks are arranged in a subwavelength periodic structure with a lattice constant (p) of 300 nm.

**Figure 2:**
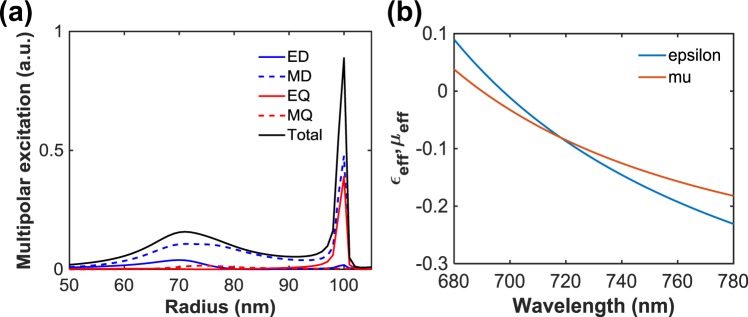
(**a**) Calculated multipolar decomposition under plane wave excitation with different disk radii and (**b**) effective medium parameters.

The diameter of the disks varies between 80 and 192 nm. We have considered a-Si material and its optical properties have been measured using an ellipsometer. The obtained data are used in our numerical investigations. The a-Si disks are fabricated on a glass substrate with a refractive index of 1.50. The deflection property of the supercell was numerically investigated using the CST Microwave Studio with the periodic boundary condition in the y-direction. Plane wave has been used as an excitation source to investigate the propagating wave response of the device. To study the resonance condition in details, multipolar decomposition of the nanodisks has been done with different disk radii shown in Fig. 2a. As can be seen, the strong resonance occurs around the disk diameter 200 nm, where the MD and EQ (electric quadrupole) are excited. However, when the disk diameters are in the range of 80 to 192 nm, the excitation of ED and MD is moderate and forms a slightly off-resonant region. This is indeed a significant advantage, leading to high transmission and low absorption, simultaneously. Alongside this, we have investigated the effective medium properties of a disk (130 nm diameter) shown in Fig. 2b. We used the Smith retrieval technique to investigate the effective medium properties of the individual nanodisks^28^.

The simulated S-parameters results were renormalised in terms of free space impedance, Z_0_ = 376.73Ω. Using the simulated S-parameters, the effective refractive index (n_eff_) and impedance (Z_eff_) of the disk can be obtained using the following equations^28^,

1 $${n}_{eff}=\pm \,(\frac{1}{kh}{\cos }^{-1}[\frac{1}{2{S}_{21}}(1-{{S}_{11}}^{2}-{{S}_{21}}^{2})]+\frac{2\pi m}{kh})$$

2 $${Z}_{eff}=\pm \,{(\frac{{(1+{S}_{11})}^{2}-{{S}_{21}}^{2}}{{(1-{S}_{11})}^{2}-{{S}_{21}}^{2}})}^{1/2}$$

where *k* = *2π/λ* is the wavenumber, *h* is the height of the nanodisk and *m* is an integer.

Using the n_eff_ and Z_eff_, the effective permittivity (*ε*_*eff*_) and permeability (µ_eff_) can be obtained easily with the following relations,

3 $${\varepsilon }_{eff}=\frac{{n}_{eff}}{{Z}_{eff}},\,\,\,\,{\mu }_{eff}={n}_{eff}{Z}_{eff}$$

It is noticeable that when the disk diameters are around 80 to 192 nm, owing to the excitation and overlap between ED and MD, an artificial magnetic response can occur by impedance matching the permittivity and the permeability of the disk. As the ED and MD are weak in this diameter region, the 2π-phase response can be achieved with high transmission. Using the effective medium approach, the phase response of the transmitted light, after passing through the nanodisk, can be obtained as^2^, $\varphi =\frac{\omega }{c}{n}_{eff}h$, where *n*_*eff*_ is the effective refractive index of the disk and *h* represent the height of the disk. The light propagates through the disks with different sizes in non-identical manner, as a result, various effective refractive indices are generated.

**Figure 3:**
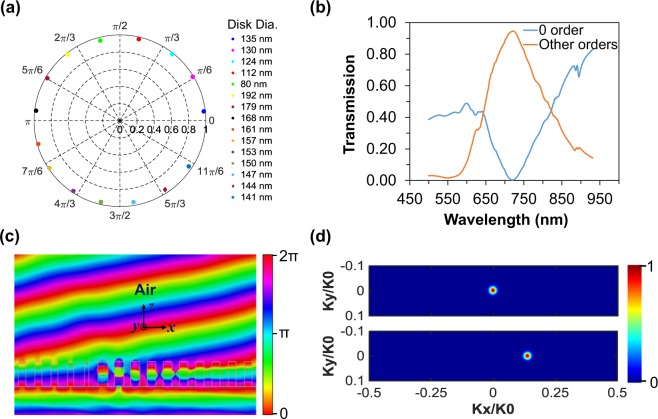
(**a**) Transmittance and phase in polar format at the operating wavelength of 715 nm. (**b**) Simulated transmittance spectra for different diffraction orders. (**c**) The simulated phase of a plane wave propagating through the metasurfaces resulting in a diffraction angle of 8.50°. (**d**) Simulated Far-field profiles of the incident light intensity (top) and transmitted light intensity (bottom).

Furthermore, by using different *n*_*eff*_, phase response from 0 to 2π can be achieved. Individual nanodisks transmission and phase response at an operating wavelength of 715 nm are depicted in Fig. 3a. It demonstrates that individual nanodisk exhibits almost near-unity transmission which lead to the high transmission as a supercell. The transmission spectra of the individual nanodisks are shown in Supplementary Information Fig. S1. It is worth noting that the strong resonances of the individual chosen nanodisks take place between 500 nm and 707 nm, whereby the largest nanodisk with 192 nm diameter exhibits the strong resonance around the wavelength of 707 nm. By arranging the 15 nanodisks in a phase increment from 0 to 2π, the full transmission spectra of the proposed gradient metasurface is shown in Fig. 3b. According to Fig. 3b, the 0^th^ order transmission of the proposed gradient metasurface is 0%, while the other orders lead to the transmittance of ~95% at the operating wavelength of 715 nm, whereby the calculated transmission efficiency is 95%. As can be seen, instead of incident light propagates straight in normal 0^th^ order, here incident light transmits to other orders, which is a clear indication of light bending.

The wave propagation through the gradient metasurface is shown in Fig. 3c. It is known that the diffraction angle of the gradient metasurface can be obtained by following the Snell’s law^18^,

4 $${n}_{t}\,\sin \,{\theta }_{t}-{n}_{i}\,\sin \,{\theta }_{i}=\frac{\lambda }{2\pi }\frac{d\varphi }{dx}$$

where *θ*_*i*_ is an incident angle and *θ*_*t*_ are the incident and diffraction light angles, respectively. The refractive indices of the surrounding media from the incident and transmitted sides are defined by *n*_*i*_ and *n*_*t*_, respectively. The operating wavelength is *λ*, and *dφ/dx* indicates the phase gradient. The individual phase of the unit cell is defined by dφ and period of the unit cell is defined by dx. According to Equation 1, by reducing the unit cell size and increasing the phase of the individual unit cell, the deflection angle can be increased significantly. Considering our 15 nanodisk supercell which provides *dφ* equals to π/7 and the dx equals to 300 nm, and according to Equation 4, our theoretically proposed fifteen nanodisks supercell provides the deflection angle of 9.80°. We have also investigated the far-field profile of the proposed gradient metasurface shown in Fig. 3d. The far-field profile of the incident light intensity is shown in the top part and the deflected beam intensity for +1 diffraction order after passing through the proposed gradient metasurface is shown in the bottom part of the figure. The calculated diffraction efficiency is 100% and the deflection efficiency is 95%, whereby both values demonstrate a clear improvement in deflection efficiency, as compared to the recently reported works^19,29^. Also, the calculated deflection angle is 8.50° $(\theta ={\sin }^{-1}({k}_{x}/{k}_{0})$).

**Figure 4:**
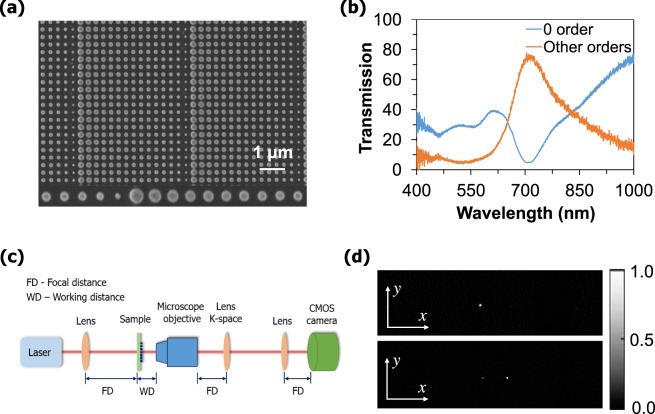
(**a**) Scanning electron microscopy (SEM) image of fabricated 15-disk supercell based metasurfaces with 90 × 90 μm^2^ total size. (**b**) Experimental measurement of transmission spectra for zero and other orders. (**c**) The schematic of the measurement set-up to investigate the beam steering by far-field analysis. (**d**) The experimental investigation of the far-field profiles of the incident light intensity (top) and transmitted light intensity (bottom) captured by a CCD.

In order to prove the concept experimentally, we have fabricated and tested the metasurface. A Scanning Electron Microscopy (SEM) image of the fabricated periodic supercell structure, consisting of fifteen nanodisks is shown in Fig. 4a. To fabricate the samples, first 352 nm a-Si layer has been deposited on a glass substrate using the plasma-enhanced chemical vapour deposition (PECVD) process. Subsequently, the proposed supercell based gradient metasurfaces have been fabricated using electron beam lithography (EBL). The total area of each gradient metasurface is 90 × 90 µm^2^. The details of the fabrication process are given below in the method section. The experimentally measured 0^th^ order transmittance of the proposed gradient metasurface is only 5% while the other orders transmittance is 78% at 715 nm (Fig. 4b). It indicates the incident light wave is deflecting after passing through the gradient metasurfaces.

The measured transmission efficiency (ratio of the transmitted light to the incident light) is 83%, which is the highest reported value as compared to the recently reported metadevices^18,22,30^. The experimental set-up of the transmission measurement can be found in Supplementary Information Fig. S2. The schematic of the experimental setup for deflection measurements is shown in Fig. 4c. The light deflects during the propogation through the sample. To ensure the capture of all the transmitted light through the sample, 100x objective has been used. Finally, a CCD camera has been utilised to capture the image of diffraction orders. The far-field profile of the incident light intensity is shown in the top part and the deflected beam intensity for +1 diffraction order after passing through the proposed metasurface is shown in the bottom part of fig. 4d. It can be seen that most of the light is transmitted to the +1 diffraction order, while the intensity at the other orders is minimal. The measured beam-deflection angle is 8.40°, which shows an excellent agreement with our simulated deflection angle, shown in Fig. 3d. Also, the measured deflection efficiency is 71%.

It is worth mentioning that deflection angle can be controlled in various ways such as changing the environment medium refractive index, angular light incident, reducing the supercell length, and increasing the phase coverage of the supercell^18^.

**Figure 5:**
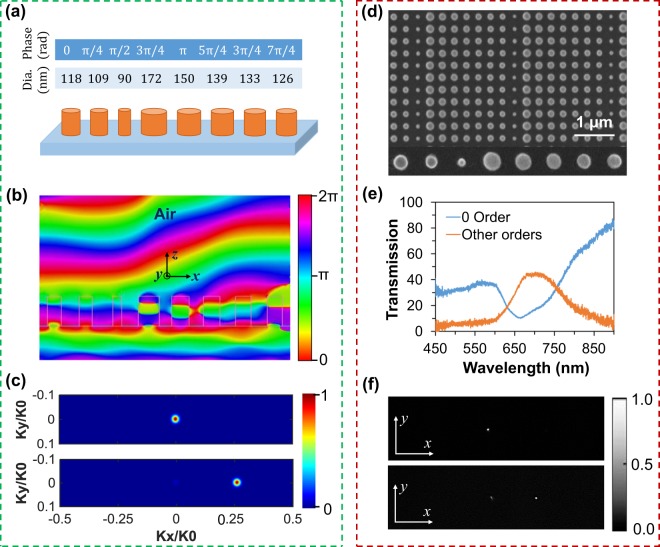
(**a**) Schematic of the smaller supercell which consists of eight nanodisks, where each responsible for phase shift from 0 to 2π with π/4 increments. (**b**) Simulated phase profile obtained by the metasurface resulting in a diffraction angle of 15.66° at 680 nm wavelength. (**c**) Simulated Far-field profiles of the incident light intensity (top) and transmitted intensity (bottom). (**d**) SEM image of the fabricated metasurface, which consists of eight nanodisks and corresponds to the phase shift 0 to 2π with π/4 increments. (**e**) Experimental measurement of transmission spectra for zero and other orders. (**f**) Experimental far-field profile of the incident (top) and transmitted intensity (bottom) captured by a CCD camera.

However, changing the embedding medium and the incident angle have a negligible effect on changing deflection angle whereas reducing supercell length or increment of phase within the same supercell length can highly increase the deflection angle. Reducing supercell length, which is equivalent to reducing the number of nanodisks, leads to a higher phase gradient of the individual unit cell that can cover 0 to 2π phase response. As a result, in line with the Snell’s law^18^, propagating wave will deflect with a large diffraction angle (see Eq. 4). Another way to increase the deflection angle is exploiting multilayer metasurfaces^31^. The 1^st^ layer will bend the incident light at a certain angle, and then the 2^nd^ layer will receive the propagating light through the 1^st^ layer as incident light and deflect it with the wider angle. By considering multiple metasurface layers, the deflection angle can be altered significantly. Here we demonstrate this effect with a shorter supercell consisting of eight a-Si nanodisks only. The schematic of the shorter supercell is shown in Fig. 5a where the nanodisks diameter varies from 90 to 172 nm. Individual nanodisks show slightly off-resonance phenomena following by effective medium properties and show the corresponding phase responses at 680 nm wavelength. The full-transmission spectrum of the individual disks is shown in Supplementary Information (Fig. S3), whereby the operating wavelength has a minimal contribution of ED and MD to achieve the near-unity transmission and 2π-phase response. 15 disks supercell has been investigated at 715 nm wavelength. However, due to fabrication imperfections of the nanodisks diameter, the operating wavelength for the shorter supercell is 680 nm. It is noticeable that individual nanodisks show near-unity transmission and each of them is responsible for phase shift from 0 to 2π with π/4 increments. By reducing the number of nanodisks the phase gradient increases, which leads to higher deflection angle. The proposed eight-disk supercell theoretically provides the deflection angle of 16.50°. By arranging the nanodisks in phase increment 0 to 2π, the propagating wave through the shorter supercell shows the larger wave bending (Fig. 5b). As can be seen in the far-field intensity of the shorter supercell, shown in Fig. 5c, incident light (top) is fully transmitted through the +1 order of diffraction (bottom) where the calculated deflection angle is 15.66°.

Increasing the phase gradient rises the deflection angle, however, it reduces the transmission and deflection efficiency. The calculated transmission, diffraction and deflection efficiencies are 87%, 83% and 72%, respectively, which are the highest values as compared to the reported silicon metadevices in the visible range^19,30^. The SEM image of the fabricated shorter supercell based gradient metasurface is shown in Fig. 5d. The experimentally measured transmittance is depicted in Fig. 5e, where the 0^th^ order shows the transmittance of only 12.5% and the other orders of 44.5% at operating wavelength 680 nm. Experimentally measured transmission efficiency of the proposed shorter supercell is 57%. However, transmission efficiency can be improved by reducing fabrication imperfections. The experimentally measured far-field intensity profile is shown in Fig. 5f. The experimental measurements are in a good agreement with the numerical simulations where the incident light intensity (top) is fully transmitted through the +1 order of diffraction (bottom), and the measured deflection angle is 15.50°. Moreover, the experimentally measured deflection efficiency is 43%.

**Figure 6:**
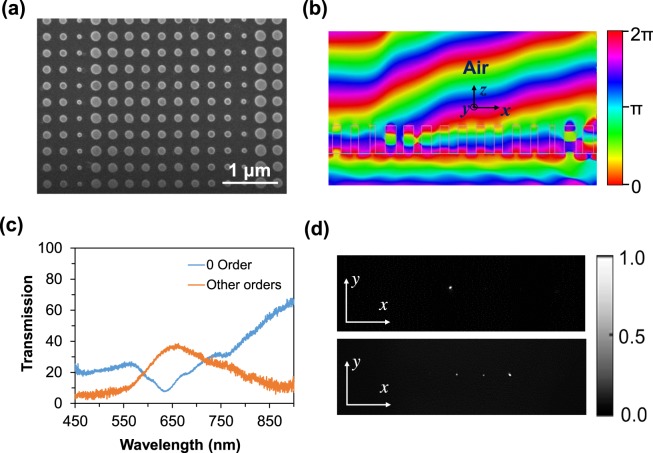
(**a**) SEM image of the fabricated 15-nanodisks supercell (3π-phase response) based metasurfaces with 90 × 90 μm^2^ total size. (**b**) Simulated wave propagation through the proposed metadevices where the nanodisks covered 0 to 3π-phase response. (**c**) Experimental measurement of transmission spectra for zero and other orders. (**d**) The experimental investigation of the far-field profiles of the incident light intensity (top) and transmitted light intensity (bottom) captured by a CCD.

Finally, we show that the deflection angle/orders of the metadevices can be controlled significantly keeping the supercell length unchanged, but, increasing the phase response of the supercell. Experimental investigation of the increasing phase response (0 to 3π) within the fifteen nanodisks supercell is shown in Fig. 6. The proposed 3π metadevice is consist of 15 a-Si nanodisks where the first eight nanodisks cover the 0 to 2π and rest of the nanodisks cover 0 to π phase profile. By alternatingly placing 8-disk unit cell and 7-disk unit cell together to design the multi-order 3π supercell metasurfaces, we combine two different beam deflectors, i.e., beam deflector made by 8-disks-unit metasurface with deflection angle *θ*_1_ and beam deflector made by 7-disks-unit metasurface with deflection angle *θ*_2_. In such a case, they can be considered as working independently and adding a transverse wave vectors *k*_1_ and *k*_2_ on the beam that illuminated at the 8-disk unit cells and 7-disk unite cells, respectively. Thus, we can divide the input beam into two parts with the proportion that is controlled by the length of the unit cell concerning to the length of the supercell. We can multiplex these two parts of the input beam and route them in different angles. Figure 6a shows the SEM image of the proposed 15 nanodisks supercell metasurface. The wave propagation of the proposed 15 nanodisks (3π phase) shown in Fig. 6b. Due to large phase gradient of 1^st^ eight nanodisk provides beam deflection angle of 14.5° while the lower phase gradient value of the remaining nanodisk bend the wave with a lower deflection angle of 8.50° which is closely matched with the theoretical deflection angle of 15.29° and 8.67°, respectively at operating wavelength of 633 nm.

Due to fabrication imperfections operating wavelength moves to the lower wavelength. The experimentally measured transmission efficiency of the proposed gradient metadevice is 43% where the 0^th^ order and other orders show the transmittance of 7% and 36%, respectively (see Fig. 6c). Experimentally investigated far-field intensity profile is shown in Fig. 6d. Due to the introduction of two different phase profile (0 to 2π and 0 to π), proposed metasurface shows light deflection not only in 1^st^ order but also 2^nd^ order as well. Here, 2^nd^ order appears due to π phase response of the last 7 nanodisks, while this 2^nd^ order is indeed the 1^st^ order of 7 disks supercell. Light passing through the 1^st^ and 2^nd^ orders are 22% and 50%, respectively with the close deflection angle of 8°. The measured deflection efficiencies are 10% and 20% of the 1^st^ and 2^nd^ diffraction orders, respectively. The findings are comparable with the reported work of nonintuitive geometry based beam splitters where two wavelengths splitter and five wavelengths splitter experimentally show the deflection efficiency of 56.4% and 17.1%, respectively at the operating wavelength of 900 nm^32^. In the proposed deflector, propagating waves coming from two different phase profile arrangement are joining correctly and deflecting. Such metadevice-based approach may have potential applications in beam multiplexing. As a result, by arranging the nanodisk from lower phase gradient to a higher phase gradient, a wave can be bend with a large deflection angle.

The performance comparison of the reported metadeflectors with the proposed one is shown in Table 1.

**Table 1:** Performance comparisons of the experimentally reported metadeflectors.

Characteristics	Disk Mode	Wavelength (nm)	Transmission Efficiency	Deflection Efficiency	Deflection Angle	Ref.
c-Si based circular nanodisk (8 unit cells)	Resonance	532	71%	67%	19.27°	^18^
poly-Si based square nanoblock (8 unit cells)	Resonance	1550	36%	—	13.1°	^19^
High-index chalcogenide based rectangular meta-atom (8 unit cells)	Resonance	5200	75%	60%	15.1°	^22^
Liquid crystal based circular nanodisks (6 unit cells)	Resonance	745	—	50%	12.0°	^23^
a-Si based circular nanodisk (8 unit cells)	Resonance	705	—	45%	10.3°	^30^
a-Si based larger supercell (15 unit cells)	Off-resonance	715	83%	71%	8.40°	This work
a-Si based shorter supercell (8 unit cells)	Off-resonance	680	57%	43%	15.50°	This work
a-Si based larger supercell (15 unit cells with 3π phase)	Off-resonance	633	43%	10%, 22%	8.0°	This work

## Conclusion

In summary, we experimentally demonstrated three different supercells-based deflection metadevices for efficient wave manipulation in the visible range. We numerically achieved the maximum transmission and deflection efficiency of 95% and 95%, respectively, for 15-disks supercell (2π phase) based gradient metasurface at operating wavelength 715 nm, while experimentally achieved transmission and deflection efficiencies are 83% and 71%, respectively. Importantly, numerically and experimentally attained deflection angles are of 8.66° and 8.40°, respectively. Moreover, by reducing the supercell length, we experimentally achieved the deflection angle of 15.50°. Furthermore, we also experimentally demonstrated that the waves coming from two different supercells can be combined and efficiently bend the propagating wave. The proposed metadevices can pave the way for high transmittance silicon metasurfaces with broad range of applications in the visible region.

## Methods

### Fabrication

We have used a thin glass substrate with 170 µm thickness for this project. Initially, the substrate was cleaned by oxygen plasma. Subsequently, 352 nm a-Si was deposited on top of the substrate using the Plasma-enhanced chemical vapour deposition (Oxford PlasmaLab System 100). The optical properties (real and imaginary part of the refractive index) of the a-Si, used in the simulations, were measured via Ellipsometer (JA Woollam M2000D). The samples were then patterned using standard electron beam lithography (Raith 150) process, followed by development and deposition of 50 nm Cr for the masking purpose. After lift-off, the Cr masks were transferred into the silicon film by using inductively coupled plasma (ICP) etching process. Finally, residual Cr was removed by Cr etchant.

### Experimental measurement

We have measured the transmission spectra of the fabricated gradient metasurfaces using a home-built white-light spectroscopy setup in a confocal configuration. The sample was illuminated from the back-side and NIR 5x and 100x objectives were used to capture the transmission spectra for 0 and other orders. The beam deflection measurement was carried out by the experimental set-up shown in Fig. 4c. A tunable femtosecond laser (pulse duration of 200 fs with the repetition rate of 80 MHz) has been used as a light source. The spectral full-width-half-maximum of the laser line is 4.4 nm. We have used the 100x objectives to collects the transmitted light through the sample. The k-space measurement environment has been established by placing a lens at the focal distance behind the objective, and another lens is placed to image the back focal plane in real space. This real and k-space measurement has been carried out by altering (keeping and removing) the lens. In the end, a CCD camera was placed at a focal distance from the real-space measuring lens to capture the far-field images.

### Numerical simulations

We numerically investigated the transmission and phase response of the individual nanodisk using rigorous coupled-wave analysis (RCWA) method^33^. RCWA is a fast response modelling method which helps to optimise the nanodisk parameters effectively. The multipolar decomposition of the individual disk has been carried out using the finite element method in COMSOL Multiphysics in the frequency domain. The CST Microwave Studio has been used to calculate the complex transmission and reflection coefficients (S-parameters) of the nanodisk which helps to obtain the permeability and permittivity of the disk. The total transmission of the supercell based gradient metasurfaces has been calculated using the CST software with unit cell boundary condition. The waveguide ports have been used to investigate the scattering responses of the device. The wave propagation through the proposed supercells was numerically investigated by using the CST Microwave Studio with the periodic boundary condition in the y-direction. Plane wave has been used as an excitation source to investigate the response of the device. The far-field profile was investigated using the field component of the propagating waves.

## Supplementary information

Supporting Information
