# Supplementary material for: High-Efficiency Visible Light Manipulation Using Dielectric Metasurfaces
Source: Sci Rep. 2019 Apr 24;9:6510. doi: 10.1038/s41598-019-42444-y (PMC6482137; doi:10.1038/s41598-019-42444-y)
Supplement: Supplementary file 1 — Supporting Information [file 41598_2019_42444_MOESM1_ESM.docx]

**Supporting Information**

**High-Efficiency Visible Light Manipulation Using Dielectric Metasurfaces**

*Rifat Ahmmed Aoni^1,^*, Mohsen Rahmani^1^, Lei Xu^2^, Khosro Zangeneh Kamali^1^, Andrei Komar^1^, Jingshi Yan^1^, Dragomir Neshev^1^, and Andrey E. Miroshnichenko^2^*

^1^Nonlinear Physics Centre, Research School of Physics and Engineering, The Australian National University, Canberra, ACT, 2601, Australia

^2^School of Engineering and Information Technology, University of New South Wales,

Canberra, ACT, 2600, Australia

*E-mail: [RifatAhmmed.Aoni@anu.edu.au](mailto:RifatAhmmed.Aoni@anu.edu.au)

**Transmission analysis of individual nanodisk for 15 a-Si based supercell:**

The larger supercell consists with 15 a-Si nanodisks where the nanodisk diameters vary between 80 nm to 192 nm. The transmission spectrum corresponding to the individual nanodisk is shown in Fig. S1. From Fig. S1, it is clearly noticeable that the chosen nanodisk shows the strong resonance before the operating wavelength 715 nm. The closest strong resonance occurs at 707 nm wavelength for the maximum larger disk diameter 192 nm. Furthermore, individual nanodisk shows almost near-unity transmission response which is one of the key criteria for the transmissive metadevices. Previously reported deflection metadevices shows minimum transmission efficiency because of their chosen individual nanodisk shows less transmission as a result overall transmission efficiency of the gradient metasurfaces also reduced significantly^1,2^. However, transmission efficiency of a metadeflector could be improved by efficiently choosing the highly transmittive meta-atoms.

Fig. S1 Transmission spectrum with varying disk diameters.

**Experimental Set-up of Transmission Measurement:**

Fig. S2 shows the schematic of experimental set-up for transmission measurement where white light source has been used. An aperture diaphragm has been used which transmits only the wave vectors that are parallel to the light path. The measured metadevices have been fabricated in an array of supercells and the total fabricated gradient metasurfaces size were 90×90 µm^2^. The samples have been illuminated from the back side, and 5x and 100x objectives have been used to capture the zero and other orders light. As our metadevice is a blazed grating, so it is important to use 100x objective to capture all the incoming light passing through the device. Otherwise, light will bend in a certain angle and all the incoming light will not be captured with smaller objectives. Finally, ocean optics visible spectrometer has been used to observe the transmission spectrum.

Fig. S2 Schematic of the experimental transmission set-up.

**Transmission analysis of individual nanodisk for 8 a-Si based supercell:**

Transmission spectrum of individual nanodisk of shorter supercell shown in Fig. S3. It is clearly visible that at operating wavelength 680 nm. By following the impedance matching phenomena, the chosen individual nanodisk shows near-unity transmission at the operating wavelength 680 nm and also corresponding phase response.

Fig. S3 Transmission spectrum with varying disk diameters.

**References**

1 Zhou, Z. *et al.* Efficient silicon metasurfaces for visible light. *ACS Photonics* **4**, 544-551 (2017).

2 Shalaev, M. I. *et al.* High-efficiency all-dielectric metasurfaces for ultracompact beam manipulation in transmission mode. *Nano Lett.* **15**, 6261-6266 (2015).
